# A Glance at Some Contemporaries

**Published:** 1915-10-16

**Authors:** 


### A GLANCE AT SOME CONTEMPORARIES.

### Extracts from Exchanges.

### The Builders again at Guy's.

Once more the builders are at work at Guy's Hospital, and we learn from the *Gazette* that a new building is being erected between the east-end of "Clinical" and the Colonnade steps, which is to serve as a waiting-room of the massage department. The dining-hall in the Nurses' Home is also being enlarged to hold forty more nurses.

### Deceptive Disinfectants.

The *Sanitary Record* calls attention to a pamphlet on the homely subject of soap, which has been compiled by a well-known firm of soap manufacturers, in which occur the following remarks: "Anything with a smell and well-sounding name is good enough for some people. One smell that can only hide another smell and cannot destroy the bacteria is a positive danger to life, as it lulls us into a sense of false security until it is too late to cope with the disease." As our contemporary suggests, purchasers of cheap and ineffective disinfectants should take this to heart.

### Fire Protection in Old Hospitals.

Dr. J. M. Peters, Superintendent of Rhode Island Hospital, has contributed an interesting article to the *Trained Nurse and Hospital Review* of New York on "Fire Protection and Prevention in Old Hospitals." Generally speaking, the methods suggested are applicable to most buildings in which there is an aggregation of individuals, and the rules respecting the disposal of combustible waste materials, the storage of inflammable liquids, the use of automatic sprinklers, the night-watch, and so on are such as are already observed in many institutions. In hospitals and other public institutions, as Dr. Peters rightly says, every consideration of designor construction must be subordinated to that of safety.

### The Abuse of Drugs.

Dr. James Burnet contributes to the *Prescriber* an article on the "Abuse of Drugs," in which he refers to the unfortunate fact that many a newly-qualified medical man is ignorant of prescription-writing, and in consequence is very apt to write prescriptions of the blunderbuss type, in which not only are unnecessary drugs included, but drugs which are therapeutically opposed to each other. He quotes a few examples of almost incredible concoctions, and draws the very proper conclusion that a knowledge of drugs, their uses, doses, and how to prescribe them, ought to be demanded of every student at his final examination in medicine. It is common knowledge that ignorance of prescribing is largely responsible for the increasing use of tablets and for the too extensive employment of German drugs.

### The Hygiene of Illumination.

Commenting on the report of the Departmental Committee on Lighting in Factories and Workshops, the *Lancet* observes that the publication of this report marks a new epoch in the history of public health in Great Britain, since it is the first time that lighting has been officially considered as a hygienic factor, taking its place by the side of heating and ventilation. It is a matter of common experience that on bright, sunny days everybody feels better than in gloomy weather, and it is not altogether surprising to find that in factories improved lighting increases both the quantity and the quality of the output of work, or that in one instance the earnings of the workers increased by more than one-tenth after the installation of a better system of lighting.

### Refusal to Submit to Operations.

Apropos of the note in a recent issue of The Hospital regarding the position of French soldiers who refuse to submit to operations, it is interesting to note that in America the question of the right of an injured workman to refuse to be operated upon has been before the Courts. The *New York State Journal of Medicine* reports a decision of the Supreme Court of New Jersey that an injured employee is not compelled to assume the risks of an operation in which death may follow, however slight that probability may be, in order that the pecuniary obligations created by the law in his favour against his employer may be minimised. This decision may be rather harsh from the point of view of the employer in this particular case, since there was medical evidence to the effect that had the employee been operated upon he would probably have recovered in about six months, but if the operation were not successful he would have been left in about the same condition as before the operation. It was properly held, however, by the Court that injuries were permanent unless relieved, and that it was wrong to proceed on the theory that the injured employee should submit to an operation against his will.

